# Sudden Changes and Their Associations with Quality of Life during COVID-19 Lockdown: A Cross-Sectional Study in the French-Speaking Part of Switzerland

**DOI:** 10.3390/ijerph18094888

**Published:** 2021-05-04

**Authors:** Manon Duay, Margot Morgiève, Hélène Niculita-Hirzel

**Affiliations:** 1 Department of Occupational Health and Environment, Centre for Primary Care and Public Health (Unisanté), University of Lausanne, CH-1066 Epalinges, Switzerland; manon.duay20@gmail.com; 2 Department of Emergency Psychiatry and Acute Care, Lapeyronie Hospital, CHU Montpellier, INSERM, Univ Montpellier, Neuropsychiatry: Epidemiological and Clinical Research, 34 000 Montpellier, France; margotmorgieve@yahoo.fr; 3 Brain and Spine Institute (ICM), Hôpital de la Pitié-Salpetrière, 75 013 Paris, France; 4 Centre for Research on Medicine, Science, Health, Mental Health, and Society (Cermes3), 75 006 Paris, France

**Keywords:** lifestyle changes, health-related quality of life (HRQoL), risk perception, COVID-19, coping, media

## Abstract

The lockdown due to the COVID-19 pandemic has led to various sudden changes in a large number of individuals. In response, the question of how individuals from different social and economic strata cope with those changes has arisen, as well as how much they have affected their mental well-being. Choosing strategies that cope with both the pandemic and the well-being of the population has also been a challenge for different governments. While a large number of studies have investigated the mental health of people from different populations during the COVID-19 pandemic, few have explored the number and type of changes experienced during lockdown by the general population, alongside their relationships with health-related quality of life (HRQoL). To fill this research gap, an observational cross-sectional study on those associations was conducted in the French-speaking part of the Swiss general population. Data were collected from 431 participants during the first four weeks of lockdown due to COVID-19. Multivariate regressions were used to identify the sociodemographic profile of the population that experienced different types and numbers of changes during this period, the association of those changes with the HRQoL—mental and physical—and infection beliefs, and the perception of the governmental measures. We show that the more changes people experienced, the lower their mental HRQoL; however, adherence to governmental measures has helped people to cope with the imposed changes, even though the number of unexpected and unwished changes have strained their mental HRQoL. The low-income population experienced financial difficulties and changes in their food intake more frequently, while dual-citizenship or non-Swiss individuals declared conflictual situations more frequently. Sport practice had a positive association with mental HRQoL; nevertheless, a decrease in sport practice was frequently reported, which correlated with a lower mental HRQoL. Risk perception of COVID-19 increased with lower physical HRQoL score, which supports the efficiency of governmental communication regarding the pandemic. Our results support that government measures should be accompanied by effective and targeted communication about the risk of infection, in order to encourage all strata of the general population to follow such measures and adapt to the changes without unduly affecting their mental health. The usage of such tools might help to reduce the impact of policy-imposed changes on the mental HRQoL of the general population, by inducing voluntary changes in informed and engaged populations.

## 1. Introduction

The impact of changes on the well-being and functioning of individuals depends on the way that they respond to and cope with those changes. Political changes, dislocations, uncertainties, lifestyle changes, or job changes have been recognized as particularly challenging for individuals [1]; however, none of these changes have affected as many people at the same time as during the social and economic lockdown which was put into place in 2020, in order to curb the COVID-19 pandemic. The crisis linked to COVID-19 has the particularity of bringing about multi-scale changes: it goes beyond the health dimension alone, and constitutes an economic, political, and social crisis. Indeed, the interactions between these different social and individual levels have further complicated the situation [2,3,4].

One of the strategies for dealing with the pandemic adopted by some governments has been to impose a partial or full lockdown, which has drastically changed the lives of most of their citizens. Those populations had to cope, in a short period of time, not only with the unpredicted changes that might occur—such as the death and disease of loved ones, conflictual situations, and job loss—but also with the imposed ones. The Swiss population was among the first—after the Wuhan region in China and the north of Italy—to suffer from such sudden imposed changes in multiple aspects of daily life: social interactions (distancing), the way of working (teleworking, partial unemployment, loss of household income), and family dynamics (closure of schools, playgrounds, leisure activities in built environments) [5]. The Swiss peculiarity lay in the very good housing conditions, the governmental support for the partial unemployment due to exceptional situations (e.g., the lockdown), and in the number of people who were able to continue to work from home (70% during the first wave of COVID-19 [6], but 41% in France in the same context [7]), which partially reflects the high per capita income. This peculiarity provided an opportunity to test different predictions on the importance of changes brought by the pandemic on the well-being of the general population.

One rapidly reported psychological reaction to the COVID-19 pandemic was a rise in anxiety or/and a mental distress in the confined general population [8,9], as well as in patients with confirmed COVID-19 or with compatible symptoms [10]. The impact of COVID-19 has greatly exceeded that of other outbreaks, such as Ebola, swine flu, or Middle-East Respiratory Syndrome (MERS) [11,12,13]. The major factors involved were the imposed social distancing and social deprivation, in particular for younger population groups [14]. Such social deprivation was a direct consequence of the governmental restrictions imposed; that is, the closure of schools and businesses [14] or forcing people to stay at home [15]. This may have resulted in a negative perception of governmental measures [16]. The magnitude of the emerging global public health problem was forewarned by the decrease in mental well-being of the general population [17]. People who were usually able to cope with stressors/changes may have been weakened by the decrease in their mental well-being, and may have become less able to cope with them during the pandemic [18]. Other identified key components of the emotional and behavioural response to the pandemic included uncertainty and fear [3]. Intolerance of uncertainty can result in anxiety [19], and can lead to maladaptive or adaptive strategies to cope with it [20]. While maladaptive strategies may cause more distress—and, sometimes, an increase in eating disorders [20,21,22]—adaptive strategies help to reduce anxiety levels and increase self-esteem [23]. The identification of such resources and their incorporation into health strategies is essential to help individuals deal with the loss of control and uncertainty and to support their acceptance and stabilization [20,23]. In the absence of such measures, the negative impact of the COVID-19 pandemic might be expressed by an increase in negative health behaviours (e.g., increases in alcohol and drug consumption) [24,25]. In contrast, measures proposed in other populations seemed to drive people to embrace positive changes during the first lockdown, such as increased leisure and activity, sport practice, and more hours of sleep, while decreasing smoking habits and junk food consumption [26,27,28].

One factor which has been suggested to influence the reactions of individuals to change is the nature of the change; that is, whether it is unexpected, imposed, or voluntary [1]. While the effects of unexpected (e.g., the death of a close relative; [29]), involuntary transition in career, retirement; [30,31]), or voluntary changes (e.g., practicing an enjoyable leisure activity; [32]) have been separately explored, complete studies on the weight of each of those types of change on mental well-being are missing. The interest in conducting such research during the COVID-19 pandemic has been supported by the independent reporting of different types of changes with contrasting effects on mental health. While the occurrence of involuntary changes (e.g., unemployment) has been associated with a negative effect on mental health [33], voluntary changes have been described as having a positive effect on it [26,27,28]. The identification of populations that have experienced multiple types of change (i.e., imposed, unexpected, and voluntary), along with the description of the weight of these changes on their mental well-being, can help health policy-makers to choose the changes on which to act first, in order to prevent the associated mental distress from becoming a mental disorder or leading to suicidal behaviour [34,35]. They can also be of used for governmental policies aimed at developing citizen responsibility and the perception of risk [36], as well as in identifying which communication method is effective for raising awareness about the spread of disease [37].

This study aims to explore the association between the sudden numerous changes, the way they were perceived (i.e., imposed by the government, or unexpected), and the mental and physical quality of life of individuals. Our main hypothesis is that the greater the number of changes experienced by the individuals and the higher the diversity of their nature (e.g., imposed, unexpected), the worse their mental quality of life. The secondary hypothesis is that government communication influences the COVID-19 risk perception and, therefore, influences the acceptance of the changes experienced by each individual, depending on their demographic characteristics and physical/mental quality of life. To test these hypotheses, three dimensions were explored: (1) the number and type of changes experienced during the first lockdown by a representative fraction of the French-speaking part of the Swiss general population; (2) the influence of risk perception of COVID-19 on lifestyle changes; and (3) the relationship between these changes and the mental and physical quality of life of the individuals.

## 2. Materials and Methods

### 2.1. Context, Study Design, and Population Recruited

The data were collected through an online anonymous survey which ran from 10 April to 6 May 2020, during the first lockdown declared in Switzerland. Switzerland was the second country in Europe (after Italy) to declare a state of emergency on 16 March 2020, following the declaration of the COVID-19—an infectious disease caused by the severe acute respiratory syndrome coronavirus 2 (SARS-CoV-2)—pandemic by the World Health Organization (WHO). The Swiss Government took severe measures on 20 March 2020, such as keeping only essential shops open and prohibiting gatherings of more than five people. Nevertheless, citizens were allowed to go out [38]. On 8 April, the reproductive number of COVID-19 dropped significantly below 1 and the number of confirmed cases and hospitalized cases decreased enough to allow a phase of easing restrictions [38], with the end of lockdown on April 29.

Electronic flyers and videos introducing the study were disseminated through the website “Mental et COVID-19: on en parle”, as well as through institutional and private social media platforms (e.g., the podcast called “Le Short”, LinkedIn, and a website of a local association). The survey was carried out online, on the EFS Platform of Unipark^®^ (Questback GmbH, Cologne, Germany), which was hosted in a BSI/ISO 27001 certified data centre owned by Datagroup Bremen GmbH. The participants IP addresses were not saved and no identifiable information was collected (e.g., the participants filled their belonging to an age class or house income category). Thus, anonymity was guaranteed, and there was no way to link participants with responses. The Vaud Ethics Committee authorized such an anonymous survey (CER-VD1.0_200604). Participants were informed of the aims, risks, and benefits of the study. Those who chose to participate provided informed consent. The survey took approximately 20 min to complete; participants were free to skip any questions they did not wish to answer and could stop participating at any time. A total of 525 participants agreed to participate to the study and all met the eligibility criteria (aged over 18 years and currently residing in the French-speaking regions of Switzerland). Of those, 431 finished the survey.

### 2.2. Measures

*Sociodemographic characteristics.* Participants provided information regarding their gender, age class (18–30, 31–40, 41–50, 51–60, more than 61 years old), marital status, number of children (none, 1, 2, or more than 3), education level, monthly family income category, employment status, citizenship (Swiss, dual citizenship, other), and pregnancy status for women (the variables are detailed in Table 1). They also provided information about how many people were living together (including children), the area of the living space, and whether there was access to a balcony or garden.

*Changes experienced.* A total of 22 items covering the major areas of life were used to collect information on the changes that might have been experienced by the participants during the four weeks preceding the survey. Only seven items explicitly asked if they were associated with the current COVID-19 situation. Each area questioned and the type of response associated are detailed in the following.

*Work (4 changes tracked)*. The participants responded with “yes” or “no” to four items concerning the occurrence of changes in their work lifestyle, the shift to teleworking, the shift to partial unemployment, and a decrease in household income due to the current COVID-19 situation. They responded by “less”, “equal”, or “more than usual” to the items concerning their workload and the current COVID-19 situation.

*Social life (1 change tracked).* The response format was a 5-point scale, ranging from “never” to “all the time”, for the question whether there had been times when the current COVID-19 situation had hindered their social life and relationships with others, including family, friends, and acquaintances.

*Home environment (2 changes tracked).* Two items were considered: one on home-schooling and one on house cleaning frequency. The participants responded with “yes” or “no”, regarding whether the children with whom they lived were home-schooled due to pandemic. They answered the item regarding house cleaning frequency with “less”, “equal”, or “more than usual”.

*Mode of transportation (1 change tracked).* Participants indicated, with “yes” or “no”, whether they had changed their transportation habits in the last 4 weeks. Information on their usual and actual transportation habits (walking, cycling, public transport, by car) were also collected.

*Leisure Activities (2 changes tracked).* Participants indicated, with “yes” or “no”, whether they had done activities that were different than usual, due to the current situation of COVID-19. They answered, with a 5-point scale ranging from “never” to “all the time” to the questions: “did you accomplish less activities than usual” and “did you have to stop doing some things”.

*Physical activity (1 change tracked).* Information on physical activity practiced was collected through two questions: one regarding how often they usually exercise for at least 20 min (never, almost never, less than once a week, once a week, twice a week, three times a week, or more) and one on the frequency of practice in the last 4 weeks.

*Food intake (1 change tracked)*. The participants indicated, using a 5-point scale ranging from “never” to “all the time”, at which frequency they had eaten more or less than usual in the past 4 weeks, due to the current situation of COVID-19.

*Traumatic events (5 changes tracked).* One item queried on the occurrence of unexpected events in the last four weeks, with no specific association to the COVID-19 situation (sickness, conflict, financial difficulties, deaths, moving out). The participants answered, with “yes” or “no”, whether:

- They had any novel sickness, any major illnesses they already had, a major illness affecting a family member close to them, an accident or injury, or if they had started caring for/helping a relative or friend;
- they had broken up with a close relative (their lover, family, friends) or had conflicts with neighbours;
- they had major financial difficulties;
- someone in their close circle died (spouse or partner, one of their children, their father or mother, a sibling, another relative or close friend) or if they had lost their house pet;
- they moved out: a voluntary move, a forced relocation, or the loss of their home; or
- none of the above events.

*Less accomplishment than participants would have liked (4 changes tracked).* Four items were formulated, in the same way (all finishing in “you would have liked to do”), to collect information on respondent’s frustration regarding different aspects of their life:

- workload (more, enough, or less than preferred),
- accomplish fewer things (always, often, sometimes, rarely, never),
- hang out more or less frequently than preferred (yes or no), and
- do physical activity more or less than preferred (yes or no).

*Quality of Life.* To evaluate the mental and physical health-related quality of life (HRQoL) of the participants, the French version of the Short Form-12 (SF12) was used [39]. The SF12 is a standard scale of mental and physical health functioning. It contains 12 items and 8 dimensions: physical functioning (PF, 2 items), role limitations due to physical health problems (RP, 2 items), bodily pain (BP, 1 item), general health (GH, 1 item), vitality (VT, 1 item), social functioning (SF, 1 item), role limitations due to emotional problems (RE, 2 items), and mental health (MH, 2 items) [40].

*Risk Perception of COVID-19.* Ten items were designed, according to the standard risk perception questionnaire developed by the Municipal Public Health Service Rotterdam-Rijnmond and the National Institute for Public Health and the Environment [41]: one on knowledge (a 4-point scale), one on the perception of seriousness of the disease (a 4-point scale), two on perception of susceptibility to the disease and the extent of anxiety (2- and 5-point scales, respectively), four on perception of efficacy (a 5-point scale ranging from “certainly not”/“never” to “most certainly”/“always”) and self-efficacy, and one on the information sources they believed (detailed in supplementary material, Table S1).

### 2.3. Statistical Analysis

Descriptive statistics—frequencies with proportions and means with standard deviations (SD)—were used to describe the profiles of respondents and the changes experienced. Multivariate regressions were used to determine which participant characteristics were associated with each change. Obvious imposed, unwished, and unexpected changes were regrouped into three dimensions and one score was created for each dimension, by summing equivalent weights for the considered items. A higher score indicates more changes experienced. The scores for the imposed changes were regrouped into five items: changes in household income, in work lifestyle (comprising shift to teleworking and partial unemployment), starting home-schooling, and in social life interactions. The score for unexpected changes was established based on the occurrence of five traumatic events: sickness, conflict, financial difficulties, death, and moving out. The score for unwished changes included the four items on those things that did not go as desired. A score was also generated for the overall number of changes experienced by each participant. Multivariate regression models adjusted for sex, age, citizenship, number of children at home, education status, employment status, and lockdown duration (in weeks) were applied, in order to identify associations of change scores with the mental or the physical HRQoL, as well as with the risk perception of COVID-19. Similar statistics were conducted to identify the association of risk perception of COVID-19 with the mental or the physical HRQoL. Multinomial logistic regression was used to test the relationship between the risk perception of COVID-19 and the four change scores, after controlling for the sociodemographic factors. All analyses were carried out using the STATA 14 software (StataCorp LLC., College Station, TX, USA).

## 3. Results

### 3.1. Representativeness of the Studied Population

The representativeness of a studied population makes it possible to generalize the findings to the entire population [42]. Consequently, we compared the sociodemographic characteristics of our population to those of the general population living in the French-speaking part of Switzerland, in terms of age group, sex, educational level, and household income. The age strata, the proportion of people with children (46%, *N* = 199), as well as the distribution of total household income among the 431 volunteers that filled out the online survey (Table 1) were found to be representative of the Swiss French-speaking general population [6]. The proportion of the studied population with a tertiary education level, the proportion of women, and the proportion of Swiss citizens was slightly higher than that reported in the general Swiss population (70% vs. 45%, 68% vs. 50%, and 90% vs. 74% only Swiss and 16% with dual citizenship vs. 75%, respectively; Federal Statistical Office 2019). Nevertheless, the professional status was representative of that reported in the general population [6]. During the study, 67% (*N* = 289) of the participants were employed (59%, *N* = 254 fully employed and 8%, *N* = 35 partially unemployed due to COVID-19 situation), 3% (*N* = 15) were unemployed, 12% (*N* = 52) had retired from working, and 12% (*N* = 52) were still at school. Only 2.5% (*N* = 11) of participants had declared to have been infected by the SARS-CoV-2 virus, corresponding to the known incidence in the Swiss French population during the survey. A total of 68% (*N* = 293) of participants could access an outdoor space, such as a balcony, although 70% (*N* = 302) of the participants lived in a flat. All of the participants living in a house had a garden. Finally, 86% (*N* = 371) of participants could see green space through their window.

### 3.2. Changes in Everyday Life during the Lockdown

The incidence of the 21 changes monitored in the studied population is illustrated in Table 2. The majority of participants (84%, *N* = 362) had experienced at least one imposed change (i.e., limitation in social interaction, consequence on the household income, home schooling, teleworking, partial employment). The 31–60 year-old participants that were employed and who had kids at school were those that had suffered the most from the imposed changes (*p* < 0.004). Indeed, as a direct result of governmental decisions, 87% (*N* = 258) of the working population—representing 60% of the overall volunteers—had to change their way of working; in particular, by shifting to teleworking. Thus, the teleworking population increased from *N* = 7 before lockdown to *N* = 195 during the study period. The participants that shifted more frequently to teleworking were those with a 3rd educational degree (55% vs. 7% and 14% for 1st and 2nd degree, respectively) and with a high house income (more than 10000 CHF/month). No significant differences between sexes were noticed. A total of 51 respondents were teleworking and also had to take charge of home-schooling their children. In general, the home-schooling concerned 43% of respondents with children (*N* = 85). Indirect social effects—with a change in the family income—affected a quarter of the participants (*N* = 108). Partial unemployment affected 8% of them (*N* = 36). The only categories of employment not affected by the partial employment were construction workers and medical professionals.

A total of 21% (*N* = 91) of participants declared a frequent or permanent change in their food intake due to the COVID-19 situation. The participants with a low income (less than 5000 CHF/month) were the most frequently concerned (*p* = 0.017).

Only 64% (*N* = 116) of participants who usually practiced sport three times a week maintained their training rhythm during the lockdown, while 19% (*N* = 17) of those who practiced less than once a week started intensive training. Men reduced their sport practice more frequently than women (*p* = 0.001). Participants without children or with one child reduced their sport practice more frequently than participants with two or more children. Furthermore, 65% of those who trained three times a week during lockdown were also those who went out the longest (i.e., more than one hour). No correlation between the change in sports practice and that in food intake was observed.

A total of 43% (*N* = 184) of respondents declared the occurrence of at least one unexpected change (death, conflict, disease, relocation, financial difficulties). A disease or a death event affected all categories of the studied population. Nevertheless, conflicts were more frequent in unemployed (22% vs. 13%, *p* = 0.02) and in dual citizenship or non-Swiss populations (24% and 22%, respectively, vs. 13% in Swiss; *p* = 0.045). Financial difficulties most frequently affected the very low-income population (28% vs. 1–9% for the others; *p* = 0.000).

A total of 91% (*N* = 392) of participants expressed having experienced at least one unwished change in their frequency of going out or practicing a physical activity, their capacity to accomplish things, or their workload. All sociodemographic categories were concerned. The only significant difference was noticed in the displeasure of being limited in their movements among people without or with one kid and those with two or more kids (79–84% vs. 68–62%; *p* = 0.009).

A total of 50% (*N* = 216) of the participants cumulated two or three kinds of changes perceived as negative (i.e., imposed, unexpected, unwished). The employed participants more frequently expressed that they had experienced an undesired change (95% vs. 82%; *p* = 0.000, R^2^ = 0.0749). In contrast, unexpected changes affected the participants with a low household income more frequently (60% for <2500 CHF/month and 53% for 2501–5000 CHF/month vs. 30–45% for the others; *p* = 0.002).

**Figure 1:**
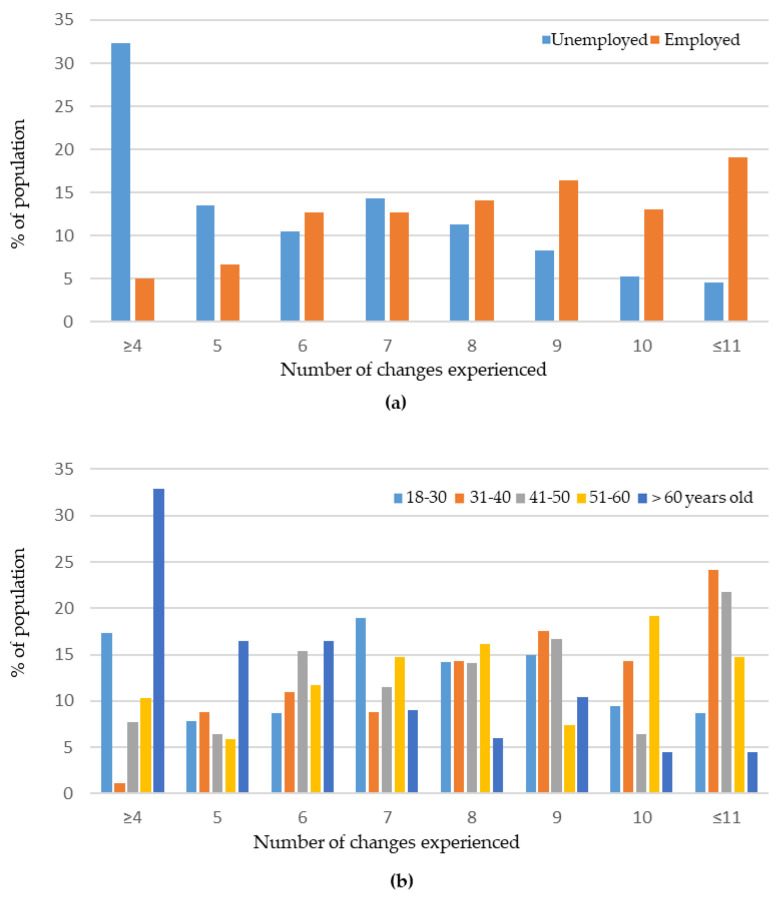
Number of changes observed in the studied population: (**a**) depending on the employment status—the incidence in the employed population is shown in orange, while that in the unemployed one is in blue; and (**b**) depending on the age class—the incidence in the 18–30 age class is shown in clear blue, that for 31–40 is shown in orange, that for 41–50 is shown in grey, that for 51–60 is shown in yellow, and that for respondents more than 61 years old is shown in dark blue.

The maximum cumulative number of changes observed were 16, with the highest frequency in the population being 7–9 changes (13%, *N* = 57; 13%, *N* = 57; and 14%, *N* = 60, respectively). A total of 82% (*N* = 353) of respondents experienced between 4 and 10 changes. A significant difference in the number of changes experienced was observed only depending on the age and employment status of the participants (Figure 1). Thus, participants over 61 years old experienced fewer changes than the others, in general, and the unemployed respondents experienced fewer changes than the employed ones. No significant difference in the total number of changes was observed, with respect to the other sociodemographic characteristics of the population.

### 3.3. Risk Perception of COVID-19

The majority of the studied population (92%, *N* = 397) defined COVID-19 as an infectious disease that can affect everyone and 89% (*N* = 384) agreed that such an infection should be avoided. A total of 53% (*N* = 228) of participants perceived COVID-19 as a severe illness and 16% viewed it as a very severe illness. People over the age of 61 were the most numerous of those who viewed the disease as very severe (35% of people who gave this answer were over 61 years old; *N* = 25), while only 10% of them (*N* = 7) were aged between 18 and 30. Considering respondents’ fear of catching the virus, 41% (*N* = 177) were slightly worried about being infected by it, 20% (*N* = 85) were worried, and 7% (*N* = 31) were very worried. People over 40 were more frequently concerned about contracting COVID-19 than younger respondents (*p* = 0.001). A total of 2.5% of the study’s participants reported to have already been infected by the virus and, therefore, were not worried about becoming infected anymore.

Participants that changed their frequency of sport practice had less tendency to consider COVID-19 as a severe or very severe illness (*p* = 0.050), while those that changed their food intake had more tendency to declare COVID-19 as a very severe illness (*p* = 0.003). Participants that had to cope with home-schooling their children (*p* = 0.020) and those that experienced conflictual situations (*p* = 0.049) declared more often that infection by SARS-CoV-2 does not need to be avoided. Participants that faced financial difficulties during the survey period declared more often that they would be highly susceptible to getting infected in the next few months if they did not take any preventive measures (*p* = 0.005). Participants that had to deal with a disease declared more often to be concerned or very concerned about being infected (*p* = 0.047).

Considering the perception of the governmental control measures, 64% of respondents thought the government’s measures were necessary and more than 67% thought that the measures would limit the spread of SARS-CoV-2; nevertheless, the respondents with a low household income (lower than 2500 or 2501–5000 CHF/month) were less likely to think that the measures would limit its spread (*p* = 0.030). A total of 72% of respondents believed that the general population has often respected the measures. Those who most often believed that the general population did not comply with the measures were in the 18–30 age bracket (*p* = 0.003), those without or with one kid (*p* = 0.003), and those with 1st or 2nd education degree (*p* = 0.000). In contrast, 97% of participants declared frequently or always respecting the measures, with no difference among age, educational degree, or number of children classes; however, a difference between sexes was observed, with women declaring it more often (*p* = 0.012).

Participants who had experienced conflictual situations reported less often that governmental action would likely or certainly limit the spread of SARS-CoV-2 (*p* = 0.011).

Television was the source which was considered the most reliable, regarding the development of the current situation, by 69% of participants, while radio was considered reliable by 32% of respondents. Participants that had to cope with a death declared more frequently relying on television (*p* = 0.045) and on radio (*p* = 0.049), while those that had changed their food intake declared less frequently to rely on radio (*p* = 0.030).

### 3.4. Association of Changes Experienced with Mental or Physical HRQoL Scores

**Figure 2:**
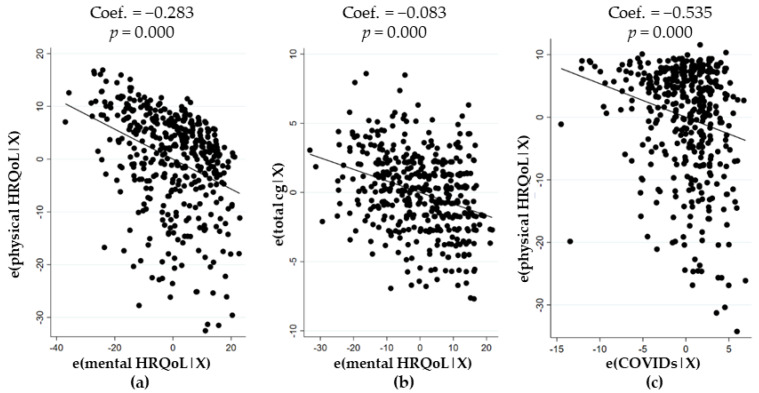
Association of mental HRQoL scores with physical HRQoL scores of participants (**a**); with the cumulative number of changes experienced (total cg) by the participants (**b**); and of the physical HRQoL score with the risk perception of COVID-19 score (COVIDs) (**c**).

The mental HRQoL score differed significantly only among age groups (*p* = 0.001). The negative correlation noticed between the mental and physical HRQoL scores of participants confirmed this observation (Figure 2a). No significant correlation with living area, occupational category, access to a green space, nationality, place of residence, number of people lived with, or type of habitation was detected. The 31–40 age class had the lowest mental HRQoL score mean (39 ± 11), while the 51–60 and 61–90 classes had the highest mean scores (45 ± 11 and 45 ± 12, respectively). The physical HRQoL score decreased with age, but increased with household income; it was higher in people with a 3rd educational degree (54 ± 8 vs. 52 ± 13 and 50 ± 12 for 1st and 2nd degrees, respectively).

Considering the changes experienced, depending on the mental and physical HRQoL score, only one was correlated with a better mental HRQoL score, while 11 were correlated with a worse mental HRQoL score (Table 2). In particular, while practicing sport during lockdown correlated with a better mental HRQoL score, only a decrease in sport practice was associated with a worst mental HRQoL score; an increase in sport practice had no significant effect on mental HRQoL score. Furthermore, people that had experienced an unexpected disease or a change in their food intake had not only a worse mental HRQoL score, but also a lower physical HRQoL score (Table 2).

An accumulation in the number and type of changes experienced was significantly associated with a decrease in the mental HRQoL score (Figure 2b; Figure S1), independently of the physical HRQoL score of participants. The number of unexpected changes explained 12% of the model and those which were unwished explained 8.6% of it (Table 2). Notice that the higher the number of unexpected changes, the worse the mental and physical HRQoL score, while the number of imposed or unwished changes seemed to lead to a high risk for the mental HRQoL of individuals (Table 2).

Concerning the overall risk perception of COVID-19, the worse the physical HRQoL score of an individual, the higher their risk perception of infection was (Figure 2c). In detail, respondents with worse mental and physical HRQoL scores were more often concerned about contracting COVID-19. Those with a lower physical HRQoL score perceived the severity of COVID-19 more and declared more often that SARS-CoV-2 infection has to be avoided (Table 3). The higher the mental HRQoL score, the more the participants believed in the efficiency of the governmental measures to limit the spread of the infection. Nevertheless, participants with a lower physical HRQoL score supported the need for the imposed governmental measures, and more often shared their thoughts that the general population did not respect these measures (Table 3).

People with a better mental HRQoL relied more frequently on TV news (*p* = 0.046), while those with a lower mental HRQoL score gave more confidence to the internet (*p* = 0.001). The participants with lower physical HRQoL scores believed the TV news (*p* = 0.010) and their social networks (*p* = 0.026) more. The number of communication channels on which the participants relied did not correlate with any of HRQoL scores.

## 4. Discussion

The semi-lockdown, decided by the Swiss government at the beginning of the COVID-19 pandemic, imposed numerous and major changes in the everyday lives of the general population, some of which were perceived as imposed or unwished. This particular situation provided us with the opportunity to test the hypothesis that dealing with multiple changes simultaneously can take a toll on the mental well-being of most people [15]. This is the first time, to the best of our knowledge, that the association of such a high number of changes (with various reasons; imposed, unwished, and unexpected) being experienced simultaneously with the mental HRQoL score of individuals, has been explored in the general population. We showed that not only the number of, but also the reason for, changes have an effect. The unexpected changes were those that most-explained the decrease in the mental HRQoL scores of respondents, followed by unwished changes. The imposed changes weighed the least on the mental well-being of the study population. Nevertheless, different sociodemographic profiles experienced unexpected or unwished changes. Low-income families and those without Swiss citizenship experienced unexpected changes more frequently, while participants with a high level of education reported unwished changes more frequently.

The most frequent unexpected changes that the low-income population had to face was financial difficulty, followed by a change in their food intake. The link between financial difficulties and food intake insecurity has been supported by a study in the population of Geneva, with two-thirds of respondents earning less than 2000 CHF; our study is in line with Bonvin et al., who showed that the loss of a job or a reduction in income can lead to lower-quality food consumption and to lower food quantity [43]. Inequalities between living standards and low-income families have also been described in the U.K. population [43,44]. Thus, the change in eating habits during the COVID-19 pandemic seemed to have been motivated more by economic than emotional reasons. Another important unexpected change, which explained 12% of the decrease in mental well-being, was the occurrence of conflictual situations. The unemployed and dual citizenship/non-Swiss populations were those that were most frequently affected. The causes of conflicts experienced by those populations could be, primarily, financial difficulties, and secondarily, differences in family beliefs, as has been shown by Prime et al. [45]. Indeed, while financial issues are a well-established risk factor for divorce, cultural, religious, and other sociological sources of variation in family beliefs (e.g., immigration and refugee history)—given their undeniable role in processes of family resilience—have also been identified as important factors [45]. Moreover, during the lockdown, the proportion of people in a bad mood increased significantly, which may also have favoured the emergence of conflicts [46].

In parallel, we researched changes that might have had a positive or protective effect on the mental well-being of the participants. Sports practice was one of the changes that has been emphasised during lockdown in different populations [24,25,47]. Our results suggested that, while starting to practice a new physical activity was not associated with an increase in the mental HRQoL, continuing sports training at the same frequency as before the lockdown had a protective effect on mental HRQoL. Nevertheless, in our population, only a few participants started or increased their physical activity, while a much larger number suffered from a decrease in the frequency of practice of a physical activity, which was associated with a worse mental HRQoL score. To our knowledge, this is the first study that has confirmed the negative impact of self-isolation and quarantine on physical activity practice, as predicted by Arora et al. [48]. In such complex scenarios, qualitative studies might bring valuable information regarding the changes having positive consequences on the well-being of people.

Imposed changes were those that weighed the least on Swiss mental well-being. Indeed, while the limitations of social interactions in the private and professional spheres negatively affected the participants, other imposed changes (e.g., teleworking, home-schooling) did not correlate with mental well-being. This finding was not explained by the lack of changes imposed on part of the population studied. These populations consisted of the medical professions and construction workers, who did not experience a shift to teleworking and whose children were not home-schooled (due to the governmental structures taking care of them). Some employees who were already teleworking without children also fell under this category. The lack of association between the shift to teleworking and mental well-being could be due to the conflicting feelings felt by some employees, who depended on the work of their colleagues. They had to deal with the difficulty of adapting to a new way of working; however, they experienced a better efficiency during teleworking. Such positive and negative characteristics, in terms of the way of working from home, have been cited in other populations during the COVID-19 pandemic [49,50]. Nevertheless, their association with the mental well-being of the employees has not been described. Another important finding of our study was that job insecurity did not seem to weigh on the mental well-being of the Swiss general population at the time of the study, in contrast to what has been observed in other populations [51]. This may have been due to the fact that the Swiss government supported partially unemployed workers, which may have had a reassuring effect on the working population. The usage of this tool seemed to pay off in protecting mental well-being.

The governmental measures were well-accepted and perceived as useful by most participants. The representativeness of our population, in terms of this way of thinking, was supported by the fast decrease in the effective reproductive number of infection three weeks after the beginning of the semi-confinement in Switzerland. The education level and the household income of respondents seemed to influence the perception of governmental measures. In addition, people with a higher level of education appeared to be more likely to practice physical distancing and to abide by the governmental measures [52]. This finding underlines the importance of adapting communication to each socioeconomic stratum of the population, in order to make as many people as possible to understand the positive consequences of adhering to governmental recommendations.

Most participants perceived the seriousness of an infection by SARS-CoV-2; in particular, respondents with worse physical HRQoL scores, those over 65 years old, and those with a co-morbidity. The risk perception of infection was similar to that recently described in an American population [53]. Thus, the Swiss governmental communication was efficient enough to make most of the people understand the risk of the infection; in particular, for those most at risk. The effectiveness of communication was supported by the positive correlation found between the public’s perception of the risk of COVID-19 and their confidence in the media that the government used to communicate through during the pandemic: television. This could be linked to the fact that they were the population at risk who had been the most targeted by the media and, therefore, who felt the most concerned by the official communication. Media influences the trust and reliance people have in the governmental measures taken, affecting communication and the response to a health crisis [54,55]. According to the WHO, risk communication and community engagement (RCCE), as an Action Plan Guidance for a good response to COVID-19, should maintain trust and prevent an overload of information [56]. Therefore, this study validates the need for clear communication during a health crisis.

Another aspect that needs to be considered in communication strategies is the fact that risk perception is directly associated with emotions, which drive and influence an individual’s judgement. Thus, the more negative emotions that individuals have, the more they will rely on negative information [57]. A study has demonstrated that emotion-focused coping strategies played a mediating role, in terms of anxiety and stress during H1N1 [19]. Strong risk communication is an important tool during a health crisis [34] and, to achieve this, an understanding of risk perception elements is necessary, trust in the governments who are communicating the information [58], and developing emotion-focused coping strategies; therefore, providing people with the opportunity to develop their critical thinking and coping skills. A mixed study collecting qualitative information on the emotions of participants in parallel to quantitative data will bring additional insight into their lived experiences. Finally, to enable people to develop strategies, public policies should increase their attention to mental health promotion for the overall population, and not only for specific parts of the population. To help them in this effort, researchers must come up with more strategies which are suited to the mental health of “healthy” and “unhealthy” populations, and test the effectiveness of these different strategies on the general population. These strategies would benefit from the integration of tools allowing individuals to develop their personal skills to manage change. In this way, people could take ownership of the acquired skills and knowledge, which will help them to feel empowered and included, as well as enabling them to respond better to imposed measures.

### Strengths and Limitations

One strength of our study was collecting information on the changes experienced by the general population in broad areas of daily life during the lockdown and assessing the weight of each type of change (i.e., imposed, unexpected, and unwished) on mental well-being. Another strength was identifying the changes chosen by the participants, based on their perception of the risk of COVID-19 and their quality of life. This methodology allowed us to discover the importance of the perception of a choice as unwished for mental well-being. To our best knowledge, this has not been described from an epidemiological point of view. In addition, we also highlighted that the weight of the imposed choices on mental well-being can be alleviated by frank governmental communication on the necessity of the measures, coupled with the proposal of supporting measures for the most affected populations.

However, certain limitations should be considered. As this survey was conducted online, populations without internet access did not have the opportunity to participate. This kind of survey is also well-known to be less frequently responded to by men, those in older age groups, those with less education, divorced and widowed respondents, and respondents on disability pension [59]. Consequently, with no surprise, we also observed this bias in our study. Another survey tool should have been used to make the study more exhaustive, such as a phone survey previously introduced by regular mail; however, this kind of survey would have taken longer to commence and would have caused us to miss our goal of capturing individual perceptions of the changes suffered in the very first weeks of the lockdown. Another limitation, imposed by the length of our questionnaire, was the use of the SF-12 to evaluate the mental well-being of individuals. Although this instrument presents only four items on mental health, which cannot provide a thorough understanding of the psychological conditions of individuals, it nevertheless allows us to compare our results with those of other studies that have used the same instrument. Using a more detailed mental health instrument would help better define the profile of the non-responding population; for example, to distinguish between those who did not participate because they were going through a mental health crisis or those who did not participate because they were going through a period of overwork (health and social workers). Larger cross-sectional studies, either conducted by phone or face-to-face, will help confirm how the number and type of changes affect the mental health of all of these different populations. In addition, a qualitative study will allow a more in-depth analysis of the perception of change at the individual level and will help to determine when some of these changes were voluntary or imposed.

## 5. Conclusions

The number of sudden changes experienced, as well as the way these changes were perceived (needed for a pandemic situation, unwished, or unexpected), weighs on the mental well-being of the general population. In the case of Switzerland, the efficient governmental communication and the rapid support proposed seemed to have softened the effect of the imposed changes on the mental well-being of the general population during the first lockdown. In contrast, the unexpected and the unwished changes affected the mental well-being of individuals. Consequently, such a distinction in the perception of the reasons for such changes may be useful to be include in further studies. Moreover, further qualitative studies are needed, in order to identify the tools to be integrated into health strategies and policies that will be most effective in helping the most vulnerable populations—low-income families and culturally mixed families—to cope with the loss of control and uncertainty. Tailoring communication to each type of population and offering tools that help individuals to develop skills (especially critical thinking skills) will enable them to take control of their own lives.

## Figures and Tables

**Table 1:** Sociodemographic characteristics of the studied population.

Variables	*N* (%)
**Gender**	
Female	291 (67.5)
**Age**	
18–30	127 (29.5)
31–40	91 (21.1)
41–50	78 (18.1)
51–60	68 (15.8)
≥61	67 (15.6)
**Citizenship**	
Swiss	320 (74.3)
dual citizenship	66 (15.3)
other	45 (10.4)
**Civil status**	
single	171 (39.7)
married or cohabiting	205 (47.6)
separated or divorced	48 (11.1)
widow or widower	7 (1.6)
**Education level**	
first level	14 (3.3)
second level	116 (26.9)
third level	301 (69.8)
**Average household income (monthly, in CHF)**	
less than 2500 CHF	25 (5.8)
between CHF 2501 and 5000	77 (17.9)
between CHF 5001 and 7500	104 (24.1)
between CHF 7501 and 10,000	92 (21.4)
between 10,000 and 12,500 CHF	63 (14.6)
≥12,501 CHF	70 (16.2)
**Number of children**	
none	232 (53.83)
one	58 (13.46)
two	88 (20.42)
≥three	53 (12.3)
**Children at home**	
yes	119 (59.8)
no	80 (40.2)

**Table 2:** Multivariate regression results of the mental and physical HRQoL score for the changes experienced.

Changes for *N* = 431	*n* (%)	Mental HRQoL	Physical HRQoL	R-Squared
		Coef.	Coef.	
Food intake (frequent or systematic change)	91 (21.1)	−0.013 ***	−0.008 ***	0.134
Different frequency in home cleaning	191 (44.3)	−0.003	−0.002	0.008
Home-schooling of children	85 (19.7)	0.000	−0.002	0.004
Stop doing things (frequently or systematically)	247 (57.3)	−0.009 ***	−0.001	0.050
Different leisure activities	341 (79.1)	0.004 *	0.004	0.012
Mode of transportation	231 (53.6)	−0.001	0.001	0.001
Different frequency in sport practice	109 (25.3)	−0.005 **	−0.001	0.017
Limitation in social interaction (frequent or systematic)	240 (55.7)	−0.014***	−0.005	0.100
Conflicts (1 or 2 events)	69 (16.0)	−0.011 ***	−0.003	0.122
Diseases (1 or 2 events)	108 (25.1)	−0.006 ***	−0.015 ***	0.104
Financial difficulties	23 (5.3)	−0.002 **	−0.001	0.016
Relocation	13 (3.0)	−0.000	0.000	0.004
Deaths	26 (6.0)	0.000	−0.001	0.003
Accomplished less things than wished	196 (45.5)	−0.015 ***	−0.004	0.118
Hanging out less than wished	325 (75.4)	−0.007 ***	−0.002	0.038
Worked more or less than wished	171 (39.7)	−0.001	0.006 *	0.017
Practiced less physical activities than wished	229 (53.1)	−0.004 *	0.000	0.012
Work lifestyle	260 (60.3)	0.006 **	0.011 ***	0.047
Teleworking	184 (42.7)	0.003	0.008 **	0.022
Consequence on the household income	108 (25.1)	−0.001	0.003	0.007
Partial employment	36 (8.3)	0.000	0.001	0.001
Number of imposed changes	360 (83.5)	−0.012 **	0.005	0.026
Number of unexpected changes	184 (42.7)	−0.021 ***	−0.021 ***	0.120
Number of unwished changes	392 (90.9)	−0.028 ***	0.001	0.086
Total number of changes	429 (99.5)	−0.083 ***	−0.01	0.111

Note: The models were adjusted for sociodemographic variables (i.e., age, sex, citizenship, education, number of children, job status, household income). * *p* < 0.05; ** *p* < 0.01; *** *p* < 0.001.

**Table 3:** Multivariate regression results of mental and physical HRQoL scores for risk perception of COVID-19.

	Mental HRQoL	Physical HRQoL	R-Squared
	Coef.	Coef.	
**What do you think the COVID-19 is?** (4-point scale on knowledge)	0.002	−0.001	0.003
**How serious do you think the COVID-19 is?** (5 point-scale from “not all” to “very serious”)	−0.002	−0.026 ***	0.100
**In your opinion, should COVID-19 be avoided?** (dichotomous outcome)	0.002	−0.004 *	0.035
**Do you think you can contract COVID-19 in the coming months if you do not take any preventive measures?** (5-point scale from “certainly not” to “most certainly”)	−0.003	−0.005	0.003
**How concerned are you about contracting COVID-19?** (5-point scale from “certainly not” to “most certainly”)	−0.019 ***	−0.038 ***	0.114
**Do you think the measures taken by the authorities during lockdown are …**			
… **necessary?** (5-point scale from “certainly not” to “most certainly”)	0.004	−0.010 *	0.020
… helping to prevent the spread of the infection? (5-point scale from “certainly not” to “most certainly”)	0.009 **	0.000	0.018
... being respected by the general population? (5-point scale from “never” to “always”)	0.006	0.009 *	0.015
**Are you able to carry out the imposed measures?** (5-point scale “never” to “always”)	−0.005	−0.005	0.009
**Risk perception of COVID-19 overall score**	−0.006	−0.081 ***	0.046

Note: The models were adjusted for sociodemographic variables (i.e., age, sex, citizenship, education, number of children, job status, household income). * *p* < 0.05; ** *p* < 0.01; *** *p* < 0.001.

## Data Availability

The data presented in this study are available on request from the corresponding author. The data are not publicly available due their partial use in the present publication.

## Supplementary Materials

The following are available online at https://www.mdpi.com/article/10.3390/ijerph18094888/s1, Table S1: Content of the questions included in the Risk Perception of COVID-19 instrument and incidence of the responses in the studied population. Figure S1: Physical and mental HRQoL score of participants colored differently depending on the number of types of changes experienced.
