# Supplementary material for: Sudden Changes and Their Associations with Quality of Life during COVID-19 Lockdown: A Cross-Sectional Study in the French-Speaking Part of Switzerland
Source: Int J Environ Res Public Health. 2021 May 4;18(9):4888. doi: 10.3390/ijerph18094888 (PMC8124785; doi:10.3390/ijerph18094888)
Supplement: Supplementary file 1 [file ijerph-18-04888-s001.zip › ijerph-1178021-supplementary.pdf]

Sudden changes and their associations with quality of life during a COVID-19 lockdown: a cross-sectional study in the French speaking part of Switzerland

Manon Duay, Margot Morgiève and Hélène Niculita-Hirzel

Supplementary materials:

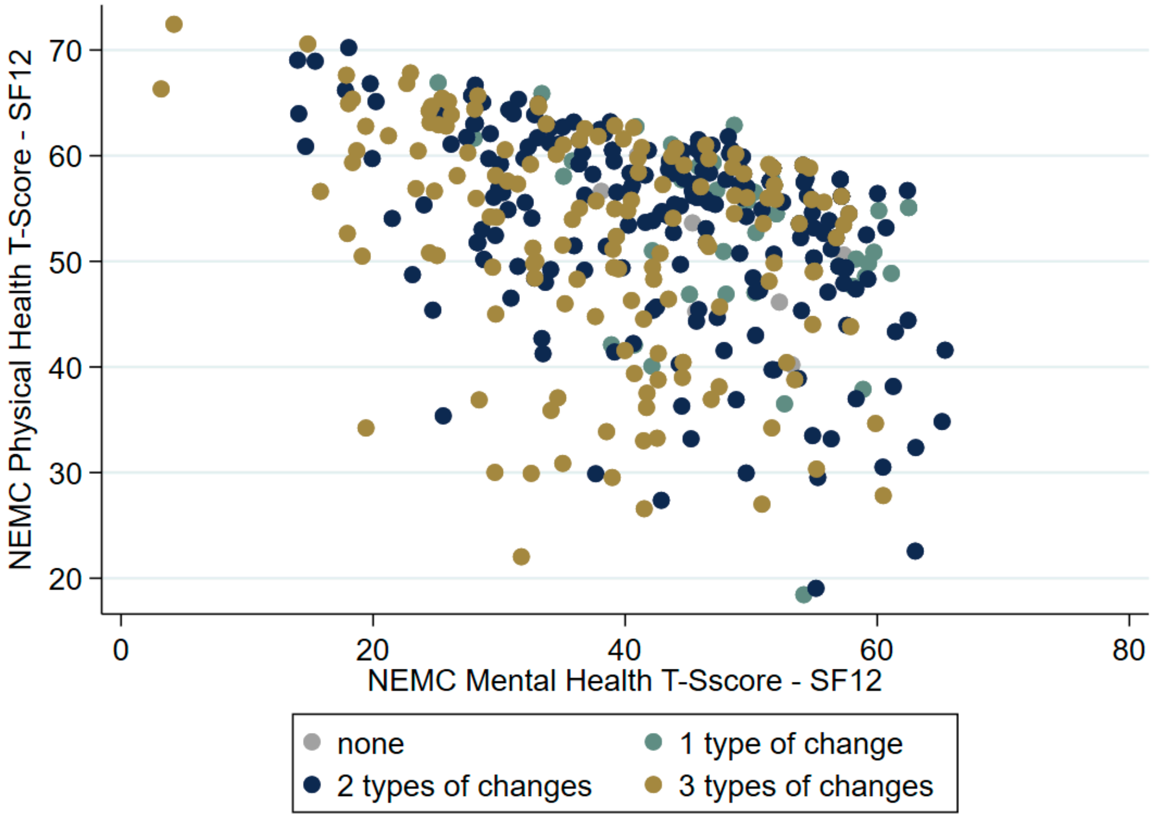

Figure S1. Physical and mental HRQoL score of participants coloured differently depending on the number of types of changes experienced.

Table S1. Content of the questions included in the Risk Perception of COVID-19 instrument and incidence of the responses in the studied population.

**C1. What do you think the Covid-19 is?**	
an infectious disease that can affect everyone	92.34%
an infectious disease that affects only frail or elderly people	6.03%
a flu	0.93%
none of it	0.70%
**C2.How serious do you think the COVID-19 is?**	
very serious	16.47%
serious	53.13%
slightly serious	26.68%
not serious	3.25%
not at all serious	0.46%
**C3.In your opinion, should Covid-19...**	
be avoided	89.33%
not be avoided	10.67%
**C4.Do you think you can contract COVID-19 in the coming months if you**	
most certainly	18.33%
probably yes	39.44%
perhaps not - perhaps yes	35.27%
probably not	3.02%
certainly not	3.94%
**C5. How concerned are you about contracting the COVID-19?**	
very concerned	7.19%
concerned	19.72%
slightly concerned	41.07%
not concerned	22.04%
not at all concerned	9.98%
**Do you think the measures taken by the authorities during lockdown are …**	
**C6...necessary?**	
most certainly	63.57%
probably yes	25.06%
perhaps not - perhaps yes	7.42%
probably not	2.09%
certainly not	1.86%
**C7... helping to prevent the spread of the infection?**	
most certainly	67.05%
probably yes	23.67%
perhaps not - perhaps yes	6.03%
probably not	2.32%
certainly not	0.93%
**C8... being respected by the general population?**	
never	0%
not quite	16.24%
occasionally	11.60%
often	71.23%
always	0.93%
**C9. Are you able to carry out the imposed measures?**	
never	0.46%
not quite	1.16%
occasionally	1.62%
often	43.16%
always	53.60%
